# Silencing miR-150 Ameliorates Experimental Autoimmune Encephalomyelitis

**DOI:** 10.3389/fnins.2018.00465

**Published:** 2018-07-10

**Authors:** Zhaolan Hu, Yanhui Cui, Xiaoqing Qiao, Xinwen He, Fang Li, Cong Luo, Shuang Wang, Changqi Li, Ruping Dai

**Affiliations:** ^1^Department of Anatomy and Neurobiology, School of Basic Medical Science, Central South University, Changsha, China; ^2^School of Life Sciences, Central South University, Changsha, China; ^3^Department of Anesthesiology, The Second Xiangya Hospital of Central South University, Changsha, China; ^4^Medical Research Center and Clinical Laboratory, Xiangya Hospital, Central South University, Changsha, China

**Keywords:** miR-150, experimental autoimmune encephalomyelitis, multiple sclerosis, T cells, spinal cord

## Abstract

MiR-150 regulates maturation and differentiation of T cells but how it functions in multiple sclerosis (MS) is unclear. In miR-150 knockout (KO) mice, we examined the effect of miR-150 deletion on disease severity of experimental autoimmune encephalomyelitis (EAE), an animal model of MS. After deleting miR-150, EAE disease severity was reduced according to clinical score. Histological staining and MBP immunofluorescence staining revealed that miR-150 deletion limited the extent of inflammatory demyelination and axonal damage in the spinal cord. Flow cytometry showed that CD3^+^, CD4^+^, and CD8^+^ T cells were increased in WT-EAE mice, but miR-150 deletion significantly reversed EAE-mediated up-regulation of CD3^+^, CD4^+^, and CD8^+^ T cells and down-regulation of CD19^+^ B cells. In addition, miR-150 deletion reduced the mRNA expression of IL-1β, IL-6, IL-17, and TNF-α in spleen and spinal cord after EAE induction. Thus, miR-150 deletion reduces EAE severity and demyelination, probably through inhibiting the activated immune response and the inflammation in the central nervous system.

## Introduction

MicroRNAs (miRNAs) are a class of short (20–25 nucleotides) single stranded molecules that regulate gene expression at the post-transcriptional level. After being transcribed in the nucleus and processing, miRNAs are transported to the cytoplasm. Single stranded RNA molecules then bind to an RNA-inducing silencing complex to form a miRNA/RISC-complex which can interact with target mRNA and effect mRNA degradation or the inhibition of protein translation. This leads to reduction of target protein synthesis (Carissimi et al., 2009). One miRNA can regulate several targets and one target can be regulated by several miRNAs, so miRNAs have multiple biological functions including cell proliferation and differentiation, as well as immune function (Carissimi et al., 2009; Anglicheau et al., 2010).

Evidence shows that miRNAs regulate immune function (Gantier et al., 2007; Rodriguez et al., 2007; Taganov et al., 2007; Xiao and Rajewsky, 2009). For example, miRNA181a is reported to contribute to the development of B cells (Liang and Qin, 2009; Kotani et al., 2010) and the miRNA-17-92 cluster can regulate T and B cell development (Ventura et al., 2008). Among miRNAs involved in the immune response, miR-150 is noted for selective expression in immature, resting B and T cells (Monticelli et al., 2005a).

Numerous studies suggest that miR-150 regulates maturation and activation of lymphocytes (Xiao et al., 2007; Zhou et al., 2007). A recent study confirmed that miR-150 controls B cell development by targeting transcription factor c-myb (Xiao et al., 2007). Subsequent studies showed that miR-150 is inversely associated with immunologic functions of activated B and T cells. miR-150 knockout (KO) mice have been shown to enhance T cell-dependent antibody responses and increased steady-state immunoglobulins (Xiao et al., 2007). miRNA 150 also affects maturation of immune cells and immune response, which is believed to suppress the cytokine signling-1 gene (Taganov et al., 2007).

The role of miR-150 in autoimmune disease in particular in multiple sclerosis (MS) is still unknown. MS is an autoimmune disease that T lymphocytes infiltrate into central nervous system (CNS) and lead to axonal demyelination of CNS. MS is thought to be initially mediated by pathogenic T-cells which against myelin antigens, followed by amplified immune attacks and ultimately results in neurodegeneration (Franciotta et al., 2008). However, B cell representing antigens, releasing cytokines and autoantibodies are vital to MS progression (Fraussen et al., 2016). Some studies suggest that miR-150 is down-regulated in MS patients (Fenoglio et al., 2011; Martinelli-Boneschi et al., 2012) and miR-150 in cell free cerebrospinal fluid is significantly greater in patients with relapsing-remitting MS compared with those who have other neurologic diseases and normal controls (Bergman et al., 2016). Changes of miR-150 in MS patients suggest an involvement in MS progression so we used miR-150 KO mice to investigate the effect of miR-150 deletion on experimental autoimmune encephalomyelitis (EAE) progression.

## Materials and Methods

### Animals

Female C57BL/6 mice (inbred strain, Animal Center, Central South University) were purchased from Laboratory Animal Co. Ltd. of Slack King (Longping Sci-tech Park, Changsha, Hunan, China) SCXK (Hunan) 2013-0004. Female miR-150 KO mice (C57BL/6 background) were purchased from Jackson Laboratory. Animals were housed with 4 to 5 per cage with a 12-h light/dark cycle at a constant temperature (22°C) and humidity-controlled (50 ± 5%) animal facility, with food and water *ad lib*. The experimental protocol was approved by the Animal Care and Use Committee of Central South University and conformed to the NIH Guide for the Care and Use of Laboratory Animals (No. 2013-99). All the animal experiments were conducted in accordance with the National Institute of Health Guide for the Care and Use of Laboratory Animals (NIH Publications No. 80-23), revised 1996, and was approved by the Animal Ethics Committee of the Third Xiangya Hospital (Changsha, China). All efforts were made to minimize suffering and the number of mice used.

### Antigen

The antigen which used to induce EAE was a synthetic peptide corresponding to region myelin oligodendrocyte glycoprotein (MOG) 35-55. The MOG35-55 (MW 2581.98; amino acid sequence MEVGWYRSPFSRV VHLYRNGK) was synthesized by Meilian Biochem, Ltd., Xian, China. The peptide was ≥99% pure (purified by HPLC), and synthesis was confirmed using mass spectroscopy.

### Characterization of miR-150 KO Mice

miR-150 KO mice was characterized by reverse-transcription polymerase chain reaction (RT-PCR). In brief, mice were sacrificed under deep anesthesia with sevoflurane and inguinal lymph node, thymus, spinal cord and spleen were harvested. Total RNA was isolated using Trizol reagent based on the company protocol (Invitrogen, United States). Reverse transcription was conducted as described by our recent study using Molony Murine Leukemia Virus Reverse Transcriptase (MMLV, Promega, United States) (He et al., 2016). PCR was performed with the primers (Forward 5′-CAAGGACAGGAACCCTTCAGCA-3′; Reverse 5′-CCATGATGCCTGGAAGACATTTC-3′). The PCR products wwere examined on agarose gel electrophoresis.

### Induction and Clinical Evaluation of EAE

Mice were randomized into four subgroups: wild type (WT) C57BL/6 mice, WT-EAE, miR-150 KO, and KO-EAE groups (*n* = 11/group). After mildly anesthetization with sevoflurane inhalation, WT-EAE and KO-EAE mice were treated in both flanks with MOG35-55 peptide (100 μg, sc) dissolved in physiological saline emulsified in an equal volume of CFA (Sigma, St. Louis, MO, United States) supplemented with 4 mg/ml *Mycobacterium tuberculosis* H37Ra (Difco Laboratories, Franklin Lakes, NJ, United States) and injected twice with pertussis toxin (200 ng, sc) given on the day of immunization and 48 h later. The same emulsion was used for re-immunization on day 7. WT and KO controls were not treated. Clinical assessment of EAE was performed daily after disease induction, as follows: (0) no disease; (1) limp tail; (2) limp tail and hind leg weakness; (3) complete hind limb paralysis; (4) forelimb and hind limb paralysis; (5) death (Gerwien et al., 2016). Clinical scores of mice were evaluated by a researcher blinded to treatment groups.

### Histological Staining

Another set of animals (*n* = 5/group) was immunized as described and sacrificed at 25 days after EAE immunization for histopathological analysis and flow cytometry. All mice were deeply anesthetized with sevoflurane inhalation and spleens were obtained for subsequent flow cytometry. After that, spinal cords (L4-L6) of mice were fixed with 4% paraformaldehyde and embedded in paraffin. Four-μm thick sections were stained with Luxol Fast Blue (LFB, 0.1%) for demyelination analysis and stained with H&E for infiltration of inflammatory cells analysis. LFB staining was used to observe the demyelination. The myelin sheath was stained in blue, whereas the areas of demyelination cannot stain with the dye, that is the white areas. We quantified the demyelination areas by the white areas in eight sections per animal at 40× magnification by using Image-Pro Plus software. The sizes of the infiltrating area of the inflammatory cells (mainly neutrophils, lymphocytes, and monocytes) were calculated by using the image pro-plus software on eight sections per animal at 40× magnification. The average area was normalized by WT group. The final data was expressed as relative inflammatory cell infiltration area and relative demyelination area.

### Immunofluorescence Assays

Four-μm thick spinal cord sections were used for immunofluorescence assays. The sections were permeabilized with 0.5% Triton X-100 in PBS for 20 min and blocked with non-fat 5% BSA in Tris-buffered saline for 60 min at 37°C, incubated with primary antibody [anti-GFAP antibody, cat no. ab7260, 1:200, Abcam, Cambridge, United Kingdom; anti-Iba-1 antibody, cat no. 019-19741, 1:1000, Wako; anti- Myelin Basic Protein (MBP) antibody, cat no. ab40390, 1:1000, Abcam] at 4°C overnight. The sections were incubated with secondary antibody [goat polyclonal secondary antibody to rabbit IgG (Alexa Fluor^®^ 488, for GFAP), cat. no. ab150077, 1:1000; goat polyclonal secondary antibody to rabbit IgG (Cy3^®^, for MBP and Iba1), cat. no. ab97075, 1:1000; Abcam] at 37°C for 1 h. The coverslips were stained with DAPI (1:2,000, SC-3598, Santa Cruz Biotechnology, Inc., Dallas, TX, United States) for 2 min at room temperature and mounted on slides using anti-fade mounting medium (Beijing Solarbio Science & Technology, Co., Ltd., Beijing, China). Immunofluorescence images were acquired using a fluorescence microscope (Nikon ECLIPSE 80i, Nikon Corporation, Tokyo, Japan). The fluorescence intensity of white matter of spinal cord in immunostaining images were quantified by using ImageJ (version 1.8, National Institutes of Health, Bethesda, MD, United States) on eight sections per animal at 40× magnification.

### Flow Cytometry Analysis of Spleen Immune Cells

Fresh spleens were minced and dissolved in PBS, filtered through a 40-μm cell strainer, and centrifuged at 350 ×*g* for 10 min at 4°C. Cells were washed with PBS and re-suspended in 2 ml PBA (∼1–5 × 10^7^ cells/ml). The suspension (50 μl) was incubated with anti-CD3 (eBioscience, San Diego, CA, United States PE-Cyanine7, 1:100), CD4 (eBioscience, San Diego, CA, United States APC/Cy7, 1:100), CD8 (eBioscience, San Diego, CA, United States APC, 1:100), CD19 (eBioscience, San Diego, CA, United States PE, 1:100) for 20 min at 4°C, and then mixed with 448 μl PBS. The suspension was transferred to BD Trucount Absolute Count Tubes (BD, New Jersey, FL). The standard of the experiment is to collect 4000 beads in every BD Trucount Absolute Count Tubes to stop counting. Cells and beads were analyzed by using a BD FacScanto II flow cytometer.

Non-specific binding of secondary antibodies was quantified, and a fluorescent signal was subtracted from values of experimental groups. Single-stained cells or OneComp eBeads (eBioscience, San Diego, CA, United States) were used for calculations. Unstained cells, GFP-expressing cells with 7-AAD, and fluorescence minus one (FMO) controls were used for cytometry and gating set up.

### Quantitative Real-Time PCR

Total RNA was extracted from the spleen and spinal cord tissues from WT mice, miR-150 KO mice, WT-EAE mice and miR-150 KO mice. The cDNA was obtained using the reverse transcription kit (Thermo Fisher Scientific, Waltham, MA, United States). Quantitative real-time PCR was performed with SYBR Green (Bio Rad) on CFX96 Touch^TM^ Deep Well Real-Time PCR Detection system (Bio-Rad Laboratories, Inc., Hercules, CA, United States). Primer sequences were as follows: IL-1β, forward 5′-GAAATGCCACCTTTTGACAGTG-3′ and reverse 5′-TGGATGCTCTCATCAGGACAG-3′; IL-6, forward 5′-CTCTGGCTTTGTCTTTCTTGTTATCTTT-3′ and reverse 5′-AGTTGTGCAATGGCAATTCTGA-3′; IL-17, forward 5′-CCTCAGACTACCTCAACCGTTCC-3′ and reverse 5′-GTGGTGGTCCAGCTTTCCCT-3′; TNF-α, forward 5′-AGGCGGTGCCTATGTCTCAG-3′ and reverse 5′-GCTCCTCCACTTGGTGGTTT-3′; GAPDH, forward 5′-CTGCCCAGAACATCATCCCT-3′ and reverse 5′-TGGTCCTCAGTGTAGCCCAAG-3′. qPCR was performed as follows: 95°C for 3 min, and 39 cycles of 95°C for 10 s and 60°C for 30 s. The experiment was repeated in triplicate. Data were processed using the 2^-ΔΔC_q_^ method.

### Statistical Analysis

Statistical analysis was performed using SPSS version 12 (SPSS, Inc., Chicago, IL, United States). Data are means ± SD. Clinical score analysis was performed with a repeated-measured ANOVA. Differences among three or more groups were compared with one-way analysis of variance with Tukey’s *post hoc* test. Statistical significance was determined as *p* < 0.05.

## Results

### Deletion of miR-150 Ameliorates Experimental Autoimmune Encephalomyelitis

**FIGURE 1:**
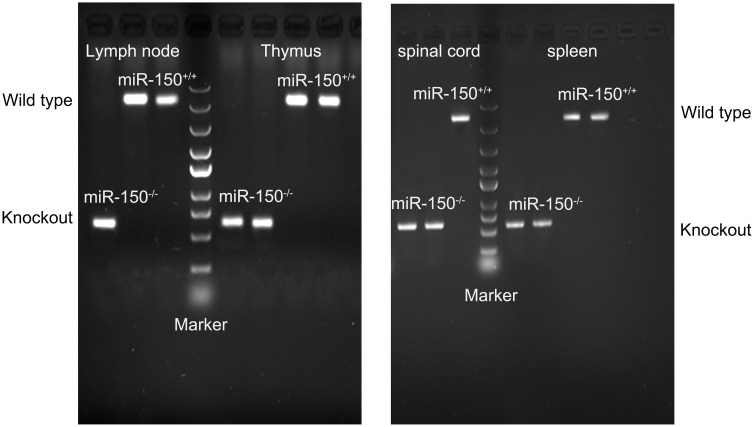
Characterization of miR-150^-/-^ (KO) mice. Lymph node, thymus, spinal cord, and spleen were harvested for RT-PCR. In the wild type (WT) mice, the expected PCR size is 866 bp; in the KO mice, the PCR size is 262 bp.

**FIGURE 2:**
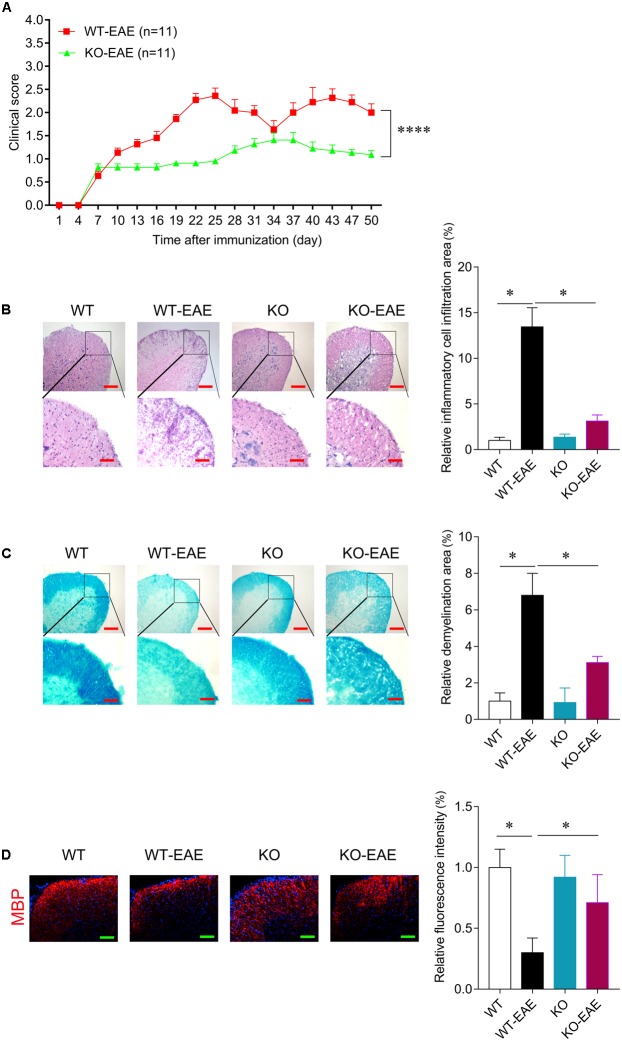
miR-150 deletion reduces disease severity of EAE. **(A)** Clinical score after EAE induction in WT and KO. In WT-EAE mice, clinical score increased at day 16 and persisted up to 50 days after immunization. The clinical score of mice in KO-EAE was significantly lower than in WT-EAE group. ^∗∗∗∗^*p* < 0.0001, WT-EAE vs. KO-EAE. **(B)** Representative images of H&E staining at day 25 after EAE induction in WT-EAE and KO-EAE mice. Upper scale bar = 200 μm, lower scale bar = 100 μm. The infiltration area of inflammatory cells in KO-EAE mice is reduced compared with WT-EAE mice. **(C)** Representative images of Luxol fast blue (LFB) staining at day 25 after EAE induction in WT-EAE and KO-EAE mice. Upper scale bar = 200 μm, lower scale bar = 100 μm. Note that demyelination in the ventral white matter of spinal cords of KO-EAE mice is reduced compared with WT-EAE mice. **(D)** Representative images of immunofluorescence staining for MBP at day 25 after EAE induction in WT-EAE and KO-EAE mice. ^∗^*p* < 0.05. Scale bar = 100 μm.

RT-PCR was used to confirm deletion of miR-150. Total RNA was extracted from lymph node, thymus, spinal cord, and spleen in the WT or miR-150 KO mice. The PCR product in miR-150 KO mice is a 262 bp fragment, while the product in WT mice is an 866 bp fragment, suggesting the successful deletion of miR-150 (**Figure 1**). The clinical score was assessed to examine the neurologic symptoms after EAE induction. There was a significant effect on time [*F*_(16,320)_ = 65.617, *p* < 0.0001], or genotype [*F*_(1,20)_ = 25.169, *p* < 0.0001] and interaction time × genotype [(*F*_(16,320)_ = 11.766, *p* < 0.0001] on EAE clinical score observed 50 days after immunization. Clinical score of mice in KO-EAE was significantly lower than in WT-EAE mice, indicating that deletion of miR-150 can inhibit the progression of EAE (**Figure 2A**). Our results from the H&E staining showed that, in the WT group, no significant infiltration of inflammatory cells was observed. In the WT-EAE group, the inflammatory cells were significantly increased compared with the WT group (*p* < 0.05). However, in the KO-EAE group, the inflammatory cells were dramatically reduced compared with the WT-EAE group (*p* < 0.05), and the infiltration area was drastically decreased (**Figure 2B**). LFB staining used to assess demyelinated lesions in white matter of spinal cords (**Figure 2C**) showed that WT-EAE mice had more myelin loss at day 25 post-EAE induction. In KO-EAE mice, demyelination was less than in WT-EAE mice (**Figure 2C**). In addition, we also observed that EAE challenge induced MBP loss in WT-EAE group compared with WT group, whereas miR-150 KO resulted in a significant increase in MBP expression in KO-EAE group compared with WT-EAE group (**Figure 2D**). These results suggest that deletion of miR-150 reduces myelin pathology after EAE challenge.

### Deletion of miR-150 Attenuates Activation of Astrocytes and Microglia in Spinal Cord

**FIGURE 3:**
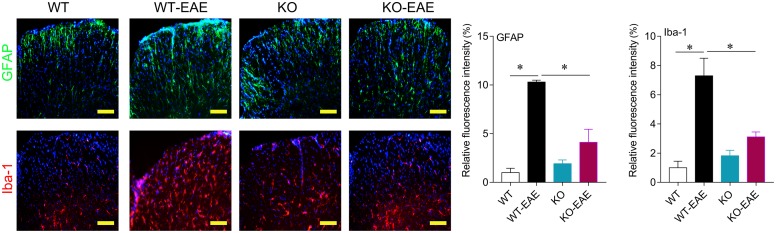
Effects of miR-150 on astrocytes and microglia activation. Representative images of immunofluorescence staining for GFAP and Iba-1 at day 25 after EAE induction in WT-EAE and KO-EAE mice. ^∗^*p* < 0.05. Scale bar = 100 μm.

Activation of astrocytes and microglia in central nerve system is an important hallmark in EAE mice. We performed immunofluorescence for GFAP and Iba-1 to evaluate the activation of astrocytes and microglia, respectively. As shown in **Figure 3**, astrocytes and microglia were intensely activated in WT-EAE mice. In the miR-150 KO mice, the expression of Iba-1 and GFAP was mild and higher than that of WT control mice suggesting the slight activation of microglia and astrocytes after deleting miR-150. After EAE immunization, microglia and astrocytes were further activated compared with KO group. However, the activation level of astrocytes and microglia was significantly lower in KO-EAE group than in WT-EAE group (**Figure 3**).

### Effects of miR-150 Deletion on T and B Cells in Spleen of EAE Mice

**FIGURE 4:**
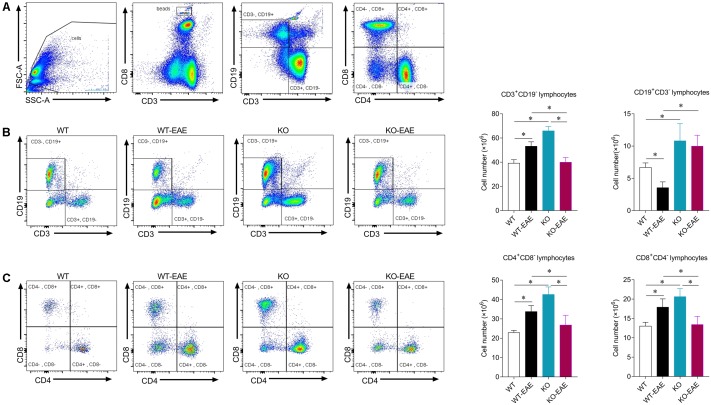
Effects of miR-150 on T and B cells in spleen of EAE mice. **(A)** Representative images of flow cytometry for beads, T and B lymphocytes in mouse spleens. Gating of mononuclear cells isolated from the spleen. **(B)** Profiles of CD3^+^ CD19^-^ and CD3^-^ CD19^+^ B cells (left) and statistical analysis (right). **(C)** Profiles of CD4^+^ CD8^-^ and CD8^+^ CD4^-^ T cells (left) and statistical analysis (right). ^∗^*p* < 0.05.

Given that CD4^+^ T cells are thought to be central to the pathogenesis of EAE and MS. We examined the effect of miR-150 deletion on expression of CD3^+^ T cells and subsets of CD4^+^, CD8^+^ T cells in the spleen at day 25 after EAE induction (Xiao et al., 2007; Jernas et al., 2013). Total cell events were counted based on the number of beads and the subsets of T and B cells were calculated within a total number of 10^6^ cells. CD3^+^, CD4^+^, and CD8^+^ T cell number was reduced in KO-EAE mice compared to WT-EAE mice (*p* < 0.001 for CD3^+^ T cell; *p* < 0.05 for CD4^+^ T cell; *p* < 0.01 for CD8^+^ T cell). Recent studies have also shown that B cells are also involved in the disease progress of EAE (Wekerle, 2017). As shown in **Figure 4**, the number of CD19^+^ B cells were down-regulated in WT-EAE compared with WT control group (*p* < 0.05). However, the number of CD19^+^ B cells was greater in KO and KO-EAE groups compared to WT-EAE or WT groups (*p* < 0.01, WT vs. KO; *p* < 0.05, WT vs. KO-EAE; *p* < 0.0001, WT-EAE vs. KO or KO-EAE, respectively).

### Effect of miR-150 Deletion on Pro-inflammatory Cytokines in EAE Mice

**FIGURE 5:**
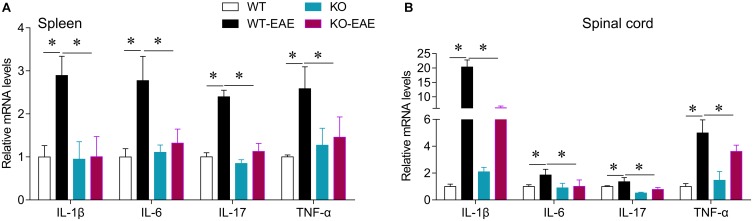
Effect of miR-150 on pro-inflammatory cytokines in EAE mice. The mRNA expression levels of IL-1β, IL6, IL-17, and TNF-α in the spleen **(A)** and spinal cord **(B)** were detected with real-time PCR. ^∗^*p* < 0.05.

To investigate the effects of miR-150 deletion on the pro-inflammatory cytokines in the EAE mice, the mRNA expression level of IL-1β, IL-6, IL-17, and TNF-α in the spleen and spinal cord was detected with real-time PCR. Compared with the WT group, the mRNA expression level of IL-1β, IL6, IL-17, and TNF-α in the spleen and spinal cord was significantly elevated in the WT-EAE group, which was significantly declined by miR-150 KO (**Figure 5**). Taken together, these results suggest that, the miR-150 deletion could significantly alleviate the elevation in pro-inflammatory cytokine levels in the EAE mice.

## Discussion

miR-150 is highly expressed in lymph nodes, spleen, and the thymus (Zhou et al., 2007). Numerous studies suggest that miR-150 regulates maturation and differentiation of T and B cells mainly via targeting c-Myb, a leucine zipper transcription factor that is key to lymphocyte development (Rose et al., 2002; Stenman et al., 2010; He et al., 2014). However, expression of miR-150 in patients with MS is inconsistent. Some studies reported no changes in miR-150 in peripheral mononuclear cells in MS patients compared with controls (Fenoglio et al., 2011), whereas other studies reported down-regulation (Martinelli-Boneschi et al., 2012; Jernas et al., 2013). Such discrepant results in clinical studies may be due to diverse individuals or different stages and treatments among MS patients, which suggested examining the role of miR-150 in MS under conditions with less variation. Here, we used miR-150 KO mice in an EAE model, a well-established animal model of MS, to verify the role of miR-150. KO-EAE mice had fewer symptoms as indicated by lower clinical scores and reduced demyelination compared with WT-EAE mice. Thus, miR-150 may exacerbate EAE disease progression.

Multiple sclerosis is a severe disease characterized by autoimmune inflammation and myelin lesions in the CNS, thought to be mediated by CD4^+^ T cell activation. miR-150 is highly expressed in immune cells and increased during development of mature T cells (Zhou et al., 2007). Thus, we investigated whether miR-150 promoted EAE progression by stimulating immune cell response. Deletion of miR-150 increased CD3^+^, CD4^+^, and CD8^+^ T cells in the spleen. Although EAE stimulation increased cell number of CD4^+^ and CD8^+^ T cells in WT mice, upregulation of CD4^+^ and CD8^+^ T cells was inhibited in KO-EAE mice. Likely, miR-150 may regulate T cell proliferation via related target gene cytokine signaling-1 (Taganov et al., 2007). In this study, our results showed that miR-150 KO without EAE challenge increased CD3^+^, CD4^+^, CD8^+^, and CD19^+^ cells number and also slightly upregulated the mRNA expression of IL-1β, IL-6, and TNF-α in spleen and spinal cord (**Figure 5**). However, the activation of astrocytes and microglia were inhibited in the KO-EAE mice as compared with WT-EAE mice of spinal cord (**Figure 3**). Although the exact mechanism is not known, the “immune tolerance” may be able to explain this discrepant phenomenon. In this regard, pre-stimulation of monocytes with lipopolysaccharide has long been known to induce tolerance, in which monocytes release less cytokines after re-stimulation by lipopolysaccharide (Fan and Cook, 2004; Netea et al., 2011; Ifrim et al., 2014). In addition, a previous study reported that pre-stimulation of T lymphocytes before the co-culture with adipose mesenchymal stem cells impairs the capacity of adipose mesenchymal stem cells to inhibit proliferation. In contrast, pre-activation of adipose mesenchymal stem cells with IFN-γ or the TLR ligand Poly I:C enhanced the ability to inhibit the proliferation of stimulated T lymphocytes (Manche-ocorvo et al., 2015). Pre-stimulation of monocytes by the cytokines GM-CSF, IFN-γ, and IL-2 significantly enhanced antibody dependent cellular cytotoxicity of monocytes. In a striking contrast, IFN-α pre-stimulation suppressed the enhancement of antibody dependent cellular cytotoxicity of monocytes (Flieger et al., 2000). Thus, it is likely that miR-150 KO may induce an immunity tolerance, leading to the attenuation of immune response, cytokine release, and glial cell activation after EAE challenge.

Evidence suggests that B cells are necessary to the progress of MS and EAE (Mann et al., 2012). B cell depletion therapy with anti-CD20 may thus be an effective approach for limiting MS (Bourdette and Yadav, 2008). miR-150 is also a key to the differentiation and maturation of B cells by targeting the transcription factor c-Myb (Monticelli et al., 2005b; Zhou et al., 2007; De Yébenes and Ramiro, 2010). B cell-specific deletion of c-Myb blocks the transition of pro- to pre-B cells (De Yébenes and Ramiro, 2010). Previous studies showed that B cells are down-regulated in spleens of EAE mice (Hayward et al., 1977; Perry et al., 1989; Mann et al., 2012). Consistent with previous studies, EAE down-regulated CD19^+^ B cells during peak EAE in WT-EAE mice in the present study. However, in KO-EAE mice, CD19^+^ B cells were more abundant compared with WT-EAE mice. Perhaps inhibition of c-Myb was lost and maturation of B cells was promoted after miR-150 deletion. Of note, some subsets of B cells such as regulatory B cells exerts protective effects on EAE or MS (Matsumoto et al., 2014). Further studies may warrant to investigate the effect of miR-150 on different subset of B cells.

Multiple sclerosis remains a challenge and current therapies suppress the immune system and have side effects (Inglese and Petracca, 2015). Alternative treatments may include disease-modifying therapies such as interferons and glatiramer acetate which may reduce MS attacks and halt disease progression with fewer adverse events (Dargahi et al., 2017). miR-150 deletion inhibited EAE disease progression and more work is required to understand how this occurred and whether this is a viable therapeutic approach for MS.

## Conclusion

miR-150 deletion reduces EAE disease severity and this may be due to inhibition of immune response, cytokine release and attenuate CNS inflammation and demyelinated lesions.

## Author Contributions

ZH conduct of the study, data collection, data analysis, and manuscript preparation and revise, final approval of the version to be published, agreement to be accountable for all aspects of the work. XQ conduct of the study, data collection, data analysis, and final approval of the version to be published, agreement to be accountable for all aspects of the work. YC and XH conduct of the study, data collection, data analysis, and final approval of the version to be published, agreement to be accountable for all aspects of the work. CL helps to do the EAE-related experiments. FL helps with data analysis. SW helps to design and analyze the data, final approval of the version to be published, and agreement to be accountable for all aspects of the work. CqL and RD are responsible for designing and interpretation of the work, data collection, data analysis, manuscript drafting and revising, final approval of the version to be published, agreement.

## Conflict of Interest Statement

The authors declare that the research was conducted in the absence of any commercial or financial relationships that could be construed as a potential conflict of interest.

## References

[B1] Anglicheau D. Muthukumar T. Suthanthiran M. (2010). MicroRNAs: small RNAs with big effects. *Transplantation* 90 105–112. 10.1097/TP.0b013e3181e913c2 20574417 PMC3094098

[B2] Bergman P. Piket E. Khademi M. James T. Brundin L. Olsson T. (2016). Circulating miR-150 in CSF is a novel candidate biomarker for multiple sclerosis. *Neurol. Neuroimmunol. Neuroinflamm.* 3:e219. 10.1212/NXI.0000000000000219 27144214 PMC4841644

[B3] Bourdette D. Yadav V. (2008). B-cell depletion with rituximab in relapsing-remitting multiple sclerosis. *Curr. Neurol. Neurosci. Rep.* 8 417–418. 10.1007/s11910-008-0064-4 18713578

[B4] Carissimi C. Fulci V. Macino G. (2009). MicroRNAs: novel regulators of immunity. *Autoimmun. Rev.* 8 520–524. 10.1016/j.autrev.2009.01.008 19200459

[B5] Dargahi N. Katsara M. Tselios T. Androutsou M. E. De Courten M. Matsoukas J. (2017). Multiple sclerosis: immunopathology and treatment update. *Brain Sci.* 7:E78. 10.3390/brainsci7070078 28686222 PMC5532591

[B6] De Yébenes V. G. Ramiro A. R. (2010). MicroRNA activity in B lymphocytes. *Methods Mol. Biol.* 667 177–192. 10.1007/978-1-60761-811-9_12 20827534 PMC3686226

[B7] Fan H. Cook J. A. (2004). Molecular mechanisms of endotoxin tolerance. *J. Endotoxin. Res.* 10 71–84. 10.1179/096805104225003997 15119998

[B8] Fenoglio C. Cantoni C. De Riz M. Ridolfi E. Cortini F. Serpente M. (2011). Expression and genetic analysis of miRNAs involved in CD4+ cell activation in patients with multiple sclerosis. *Neurosci. Lett.* 504 9–12. 10.1016/j.neulet.2011.08.021 21875645

[B9] Flieger D. Spengler U. Beier I. Sauerbruch T. Schmidt-Wolf I. G. (2000). Prestimulation of monocytes by the cytokines GM-CSF or IL-2 enhances the antibody dependent cellular cytotoxicity of monoclonal antibody 17-1A. *Z. Gastroenterol.* 38 615–622. 10.1055/s-2000-7511 11031784

[B10] Franciotta D. Salvetti M. Lolli F. Serafini B. Aloisi F. (2008). B cells and multiple sclerosis. *Lancet Neurol.* 7 852–858. 10.1016/S1474-4422(08)70192-3 18703007

[B11] Fraussen J. De Bock L. Somers V. (2016). B cells and antibodies in progressive multiple sclerosis: contribution to neurodegeneration and progression. *Autoimmun. Rev.* 15 896–899. 10.1016/j.autrev.2016.07.008 27396817

[B12] Gantier M. P. Sadler A. J. Williams B. R. (2007). Fine-tuning of the innate immune response by microRNAs. *Immunol. Cell Biol.* 85 458–462. 10.1038/sj.icb.7100091 17621315

[B13] Gerwien H. Hermann S. Zhang X. L. Korpos E. Song J. Kopka K. (2016). Imaging matrix metalloproteinase activity in multiple sclerosis as a specific marker of leukocyte penetration of the blood-brain barrier. *Sci. Transl. Med.* 8:364ra152. 10.1126/scitranslmed.aaf8020 27831901

[B14] Hayward J. L. Carbone P. P. Heuson J. C. (1977). Assesment of response to therapy in advanced breast cancer. *Cancer* 7 1–11. 10.1038/bjc.1977.42 PMC2025288 856236

[B15] He L. Xu J. M. Li H. Zhong F. Liu Z. Li C. Q. (2016). Moderate hypothermia increased the incidence of delayed paralysis through activation of the spinal microglia in an aortic cross-clamping rat model. *Int. J. Cardiol.* 220 454–461. 10.1016/j.ijcard.2016.06.169 27390969

[B16] He Y. Jiang X. Chen J. (2014). The role of miR-150 in normal and malignant hematopoiesis. *Oncogene* 33 3887–3893. 10.1038/onc.2013.346 23955084

[B17] Ifrim D. C. Quintin J. Joosten L. A. Jacobs C. Jansen T. Jacobs L. (2014). Trained immunity or tolerance: opposing functional programs induced in human monocytes after engagement of various pattern recognition receptors. *Clin. Vaccine Immunol.* 21 534–545. 10.1128/CVI.00688-13 24521784 PMC3993125

[B18] Inglese M. Petracca M. (2015). Therapeutic strategies in multiple sclerosis: a focus on neuroprotection and repair and relevance to schizophrenia. *Schizophr. Res.* 161 94–101. 10.1016/j.schres.2014.04.040 24893901

[B19] Jernas M. Malmestrom C. Axelsson M. Nookaew I. Wadenvik H. Lycke J. (2013). MicroRNA regulate immune pathways in T-cells in multiple sclerosis (MS). *BMC Immunol.* 14:32. 10.1186/1471-2172-14-32 23895517 PMC3734042

[B20] Kotani A. Harnprasopwat R. Toyoshima T. Kawamata T. Tojo A. (2010). miRNAs in normal and malignant B cells. *Int. J. Hematol.* 92 255–261. 10.1007/s12185-010-0633-6 20614203

[B21] Liang T. J. Qin C. Y. (2009). The emerging role of microRNAs in immune cell development and differentiation. *APMIS* 117 635–643. 10.1111/j.1600-0463.2009.02520.x 19703123

[B22] Manche-ocorvo P. Menta R. Del R. B. Franquesa M. Ramírez C. Hoogduijn M. J. (2015). T lymphocyte prestimulation impairs in a time-dependent manner the capacity of adipose mesenchymal stem cells to inhibit proliferation: role of interferon γ, poly I:C, and tryptophan metabolism in restoring adipose mesenchymal stem cell inhibitory effect. *Stem Cells Dev.* 24 2158–2170. 10.1089/scd.2014.0508 26058889

[B23] Mann M. K. Ray A. Basu S. Karp C. L. Dittel B. N. (2012). Pathogenic and regulatory roles for B cells in experimental autoimmune encephalomyelitis. *Autoimmunity* 45 388–399. 10.3109/08916934.2012.665523 22443691 PMC3639139

[B24] Martinelli-Boneschi F. Fenoglio C. Brambilla P. Sorosina M. Giacalone G. Esposito F. (2012). MicroRNA and mRNA expression profile screening in multiple sclerosis patients to unravel novel pathogenic steps and identify potential biomarkers. *Neurosci. Lett.* 508 4–8. 10.1016/j.neulet.2011.11.006 22108567

[B25] Matsumoto M. Baba A. Yokota T. Nishikawa H. Ohkawa Y. Kayama H. (2014). Interleukin-10-producing plasmablasts exert regulatory function in autoimmune inflammation. *Immunity* 41 1040–1051. 10.1016/j.immuni.2014.10.016 25484301

[B26] Monticelli S. Ansel K. M. Lee D. U. Rao A. (2005a). Regulation of gene expression in mast cells: micro-rNA expression and chromatin structural analysis of cytokine genes. *Novartis Found. Symp.* 271 179–187. 10.1002/9780470033449.ch14 16605135

[B27] Monticelli S. Ansel K. M. Xiao C. Socci N. D. Krichevsky A. M. Thai T. H. (2005b). MicroRNA profiling of the murine hematopoietic system. *Genome Biol.* 6:R71. 10.1186/gb-2005-6-8-r71 16086853 PMC1273638

[B28] Netea M. G. Quintin J. Jw V. D. M. (2011). Trained immunity: a memory for innate host defense. *Cell Host Microbe* 9 355–361. 10.1016/j.chom.2011.04.006 21575907

[B29] Perry J. C. Luborsky L. Silberschatz G. Popp C. (1989). An examination of three methods of psychodynamic formulation based on the same videotaped interview. *Psychiatry* 52 302–323. 10.1080/00332747.1989.11024452 2772089

[B30] Rodriguez A. Vigorito E. Clare S. Warren M. V. Couttet P. Soond D. R. (2007). Requirement of bic/microRNA-155 for normal immune function. *Science* 316 608–611. 10.1126/science.1139253 17463290 PMC2610435

[B31] Rose D. M. Han J. Ginsberg M. H. (2002). α4 integrins and the immune response. *Immunol. Rev.* 186 118–124. 10.1034/j.1600-065X.2002.18611.x 12234367

[B32] Stenman G. Andersson M. K. Andren Y. (2010). New tricks from an old oncogene: gene fusion and copy number alterations of MYB in human cancer. *Cell Cycle* 9 2986–2995. 10.4161/cc.9.15.12515 20647765 PMC3040924

[B33] Taganov K. D. Boldin M. P. Baltimore D. (2007). MicroRNAs and immunity: tiny players in a big field. *Immunity* 26 133–137. 10.1016/j.immuni.2007.02.005 17307699

[B34] Ventura A. Young A. G. Winslow M. M. Lintault L. Meissner A. Erkeland S. J. (2008). Targeted deletion reveals essential and overlapping functions of the miR-17˜92 family of miRNA clusters. *Cell* 132 875–886. 10.1016/j.cell.2008.02.019 18329372 PMC2323338

[B35] Wekerle H. (2017). B cells in multiple sclerosis. *Autoimmunity* 50 57–60. 10.1080/08916934.2017.1281914 28166681

[B36] Xiao C. Calado D. P. Galler G. Thai T. H. Patterson H. C. Wang J. (2007). MiR-150 controls B cell differentiation by targeting the transcription factor c-Myb. *Cell* 131 146–159. 10.1016/j.cell.2007.07.021 17923094

[B37] Xiao C. Rajewsky K. (2009). MicroRNA control in the immune system: basic principles. *Cell* 136 26–36. 10.1016/j.cell.2008.12.027 19135886

[B38] Zhou B. Y. Wang S. Mayr C. Bartel D. P. Lodish H. F. (2007). miR-150, a microRNA expressed in mature B and T cells, blocks early B cell development when expressed prematurely. *Proc. Natl. Acad. Sci. U.S.A.* 104 7080–7085. 10.1073/pnas.0702409104 17438277 PMC1855395

